# On the Power of Geometry over Tetrel Bonds

**DOI:** 10.3390/molecules23112742

**Published:** 2018-10-24

**Authors:** Ephrath Solel, Sebastian Kozuch

**Affiliations:** Department of Chemistry, Ben-Gurion University of the Negev, Beer-Sheva 841051, Israel; ephrath@post.bgu.ac.il

**Keywords:** tetrel bond, σ-hole, DFT

## Abstract

Tetrel bonds are noncovalent interactions formed by tetrel atoms (as σ-hole carriers) with a Lewis base. Here, we present a computational and molecular orbital study on the effect of the geometry of the substituents around the tetrel atom on the σ-hole and on the binding strengths. We show that changing the angles between substituents can dramatically increase bond strength. In addition, our findings suggest that the established Sn > Ge > Si order of binding strength can be changed in sufficiently distorted molecules due to the enhancement of the charge transfer component, making silicon the strongest tetrel donor.

## 1. Introduction

Hole interactions [1] are a relatively newly coined term that unites all noncovalent interactions in which a region of positive electrostatic potential on one atom, the hole, interacts with an electron donor. These can be based on σ, π, or δ holes depending on their type of covalent orbital origin [2]. σ-holes are formed at approximately 180° to a σ covalent bond, with the magnitude of the positive electrostatic potential depending on the electronegativity of the neighboring atoms. These interactions are further classified according to the σ-hole-bearing atom: the most studied interaction is hydrogen bonding, but there is also the widely researched halogen bonding [3,4,5], chalcogen bonding [6,7,8] (for Group VI atoms), pnictogen bonding [9,10,11] (Group V), tetrel bonding [12,13] (Group IV), and even aerogen bonding [14] (Group VIII). Better understanding of such noncovalent interactions can help in the study and future design of novel supramolecular complexes, catalysts, and crystal engineering.

Herein, we focus on the effect that the angles around the atom have on the binding strengths of tetrel bonds. We analyze this by examining the effect on the electrostatic hole and on the frontier orbitals in order to explain the dramatic changes in complexation energies. These effects were previously observed in a survey of the Cambridge Structural Database [12]: carbon demonstrates almost nonexistent σ-holes relative to Si, Ge, and Sn [13], with the only found crystal structures exhibiting σ-hole interactions with C based on three-membered rings [15] or cubanes [16], in which the angles around the carbon are far from the optimal tetrahedral angle. The effect of the angles between covalent bonds on interaction strength was also computationally explained by showing that smaller rings cause the σ-hole to be more exposed, increasing its electrostatic potential [12].

It should be noted that upon binding with a Lewis base, there is geometrical deformation around the tetrel atom as substituents move to make more room for the electron donor [17], a distortion that was computed to be more energetically costly for smaller atoms. Freezing the monomer in the complex’s distorted geometry eliminates the deformation energy and results in an increase in the tetrel interaction energy. Our aim in this study is to understand the effect of the molecular geometry on bond energy beyond such binding-caused distortions by applying molecular orbital theory on the bonding patterns.

## 2. Results and Discussion

**Scheme 1:**
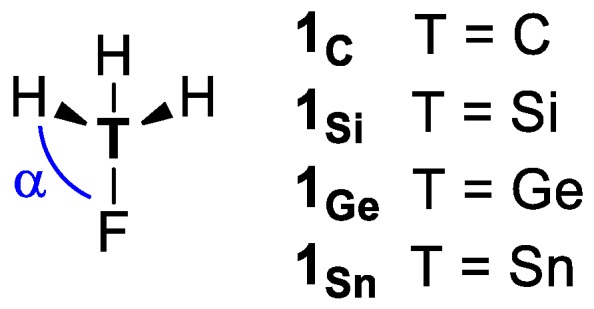
**1_T_** model systems.

To estimate the effect the substituent angles have on the σ-hole and on the shape of the frontier orbitals, we examined the TH_3_F systems (**1_T_**, with T = C, Si, Ge, and Sn, Scheme 1). A weak σ-hole may be formed at the extension of the T-H bonds, but evidently the σ-hole corresponding to the T-F bond is the dominant one. We considered the optimized structures (*C*_3*V*_) with no constrains or at three different fixed F-T-H angles (α = 109°, 100°, and 90°). All computations were performed at the MN15/Def2-TZVPD [18,19] level of theory with Gaussian16 [20] (see the Methods section).

**Figure 1:**
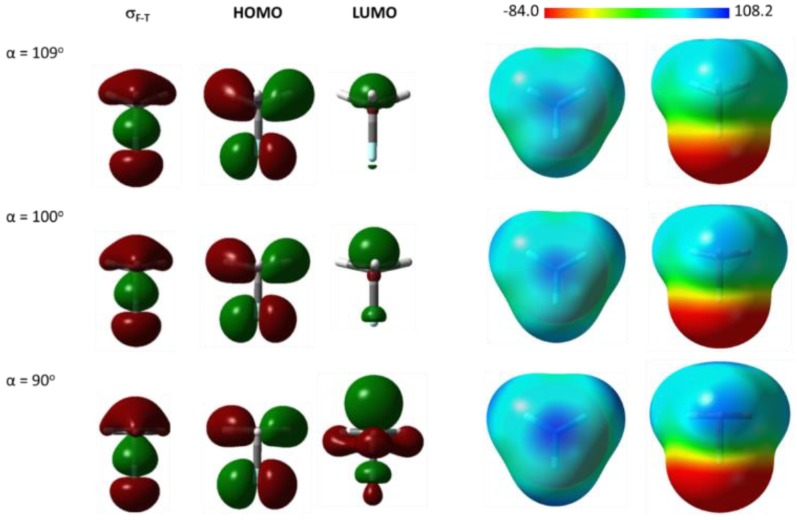
Chosen MOs and electrostatic potential (ESP) maps for **1_C_** with three different F-C-H angles (α). ESP maps are on the 0.001 density isosurface. The color scale is in kJ mol^−1^. σ_F-T_ corresponds to the bonding F-T σ orbital, irrespective of its position compared to other orbitals. The HOMO is doubly degenerate.

As can be seen in Figure 1, the LUMO for **1_C_** is an antibonding F-C σ*. This orbital mostly resides at the extension of the F-T bond (as in all **1_T_** molecules, see Figures S1–S3) and forms interactions with Lewis bases by charge transfer. For all **1_T_** molecules, the LUMO shows a larger lobe at the extension of the T-F bond as the angle decreases, which, in principle, aids the orbital interaction with the nucleophile.

The σ_F-T_ orbitals (typically, the HOMO-2) are expected to match the areas with higher and lower electron density [2,21]. In **1_C_**, the electrostatic hole did not match this criterion. At smaller α, the outer lobe on C was slightly larger, although the electrostatic potential was more positive (Figure 1 and Table 1). For **1_Si_**, **1_Ge_**, and **1_Sn_**, due to the larger and more electropositive tetrel atom, the σ_F-T_ was more localized on the F and more affected by the hydrogens. Thus, there was somewhat less electron density at the outer lobe of σ_F-T_ at smaller angles, which, in principle, matches the trend in the V_s,max_ (the maximum positive potential on the electrostatic potential (ESP) isosurface; see Figure 1 and Table 1). However, it seems that the HOMOs (σ_H-T_ orbitals) are the ones mostly responsible of taking out the electron density, enhancing the σ-hole as the angle decreases by moving the T-H away from the F-T axis (matching the V_s,max_ trend—see Table 1—and the ESP maps—Figure 1 and Figures S1–S3).

As can be expected, the F-T-H angle modifies the degree of *sp* hybridization of different orbitals. In NH_3_ [22], the frontier MOs of the planar geometry exhibit pure *s* or *p* orbitals on the nitrogen, which then mix as the angle of pyramidalization increases. Similarly, according to the NBO analysis in **1_T_**, the tetrel component of the σ_F-T_ MO has a higher *p* character when the angle is 90°, which decreases when the α angle increases (opposite to the *s* character; see Table 1). This causes stronger σ_F-T_ (shorter F-T bond length) with larger α by focalizing the lobe into the fluorine’s direction (Table 1), while also marginally reducing the outer lobe in **1_C_**, as explained above (Figure 1).

We computed the complexes of **1_T_** with HCN, a prototypical Lewis base for hole interactions that minimizes the influences coming from atoms and bonds other than the tetrel bond (ammonia, for example, exhibits attraction between its partially positive hydrogens and the partially negative hydrogens on the tetrels). The geometry parameters, dissociation energies ($D_{e}=E_{1_{T}}+E_{HCN}-E_{1_{T}\cdots NCH}$), and NBO charge transfer energies (i.e., the *n* → σ* perturbational stabilization energy, *E*^2^) are presented in Table 2.

**Figure 2:**
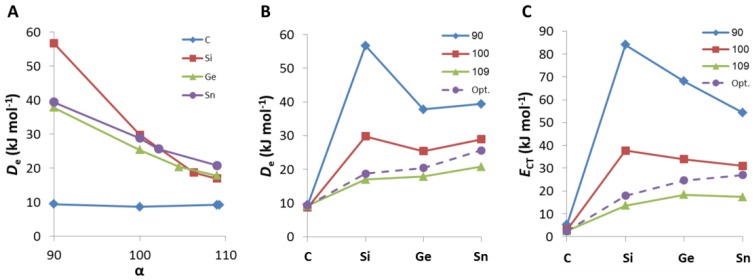
Complexation of **1_T_** with HCN: (**A**) dissociation energy as a function of the α angle; (**B**) dissociation energy as a function of the tetrel atom; (**C**) NBO *n* → σ* charge transfer energy as a function of the tetrel atom.

Table 2 shows that the T-F bonds are all longer compared to the free molecules, as expected upon interaction of a Lewis base with the σ* orbital. Bond strength, due to the higher polarizabilities of the heavier tetrel atoms, is **1_C_** << **1_Si_** < **1_Ge_** < **1_Sn_** for the fully optimized molecules or for α = 109°. For the smaller α angles, the T···N distance is shorter and the binding energies larger compared to the unconstrained systems (except for **1_C_**, which at any rate exhibits very weak binding), with the largest changes with respect to the angle observed with **1_Si_** (see Figure 2A). If we check the effect of changing the tetrel atom at each fixed α angle (Figure 2B), we can see that Ge and Sn show stronger binding than Si only for the fully optimized geometry and for α = 109°. However, upon reduction of the angle to 100° and 90°, the Si shows higher binding than the other tetrel atoms, with significantly shorter T···N distances. As can be seen in Table 2, for α = 90° the T···N distances for **1_Si_**, **1_Ge_**, and **1_Sn_** come close to the sum of the covalent radii of T and nitrogen, pointing to a more covalent character (in the extreme case of **1_Si_**, bond length is only 116% compared to the sum of the covalent radii). In addition, the NBO *n* → σ* component grows as Si > Ge > Sn for the smaller α angles (Figure 2C). This suggests that there are two competing factors affecting binding strength: polarizability, which increases upon descending the column, increasing electrostatic interactions; and orbital interactions, which are stronger for smaller atoms, except for C, and become more dominant at shorter distances and smaller α angles.

We plotted the dissociation energy of the **1_T_**⋯NCH complexes as a function of both the NBO *n* → σ* charge transfer energy and the V_s,max_ of the uncomplexed tetrel molecules (see Figure S4), which correlate, respectively, with the orbital and electrostatic interactions. The graphs show a linear relationship, indicating that the charge transfer component and the electrostatic interaction (connected with the virtual and occupied MOs of the hole bearer, respectively) go hand in hand in hole interactions [2,21]. However, there is one clear outlier in the V_s,max_ graph (Figure S4B) corresponding to **1_Si_** at 90°, for which the electrostatic potential is an insufficient descriptor. This would suggest that, for complexes with stronger binding energies and smaller intermolecular distances, orbital interaction is more significant—a sign of an incipient covalent bond.

Our results clearly show that at small angles there is a departure from the expected binding order of Sn > Ge > Si > C, as the **1_Si_** shows strongest binding for angles of 100° and 90°. This comes as a result of the better interaction between silicon and nitrogen orbitals compared to the larger Ge and Sn. However, an alternative way to look at this is to check the energy needed to distort the molecules before and after complexation [11]. For α = 100° and 90°, the distortion energy of the monomer is almost always larger than for the complex (Table S1). The difference in distortion energies (ΔE_dist_) is particularly large for Si (38 kJ mol^−1^ at α = 90°). As the difference in distortion energies equals the difference in dissociation energies (ΔE_dist_ = D_e_ constrained − D_e_ optimized), this can also explain the dramatic increase of complexation energy for **1_Si_** at these angles.

**Scheme 2:**
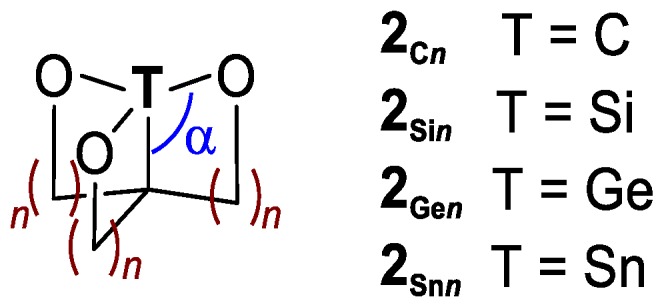
**2_T*n*_** systems.

So far, previous observations suggest that the geometry around the tetrel atom can have significant influence on the strength of the tetrel bonds, leading to unusually strong bonds at small angles, especially for T = Si. In order to check this trend in more realistic molecular models, we studied the **2_T*n*_** molecules (Scheme 2, with a C_3_ symmetry). Here, each α angle depends on the varying size of the rings determined by the number of carbon links (*n*). In **2_T*n*_**, the σ-hole is at the extension of the T-C bond and not of the T-O bonds, but the magnitude of the central hole is enlarged by the oxygens (with methylenes instead of oxygens, the σ-hole was smaller and similar in magnitude to the holes on the methylene hydrogens). Many alkoxysilanes and alkoxygermanes are known, and there is also an experimental example similar to **2_Si2_** in which the Si forms strong interactions with electron donors [23].

As can be seen from Table 3, **2_C*n*_** does not show binding when *n* = 2 or 3, and only a decrease in the C-C-O angle to 97° (*n* = 1) produces some very weak binding. For heavier tetrels, as the number of links (and, correspondingly, α) decreases, the dissociation energy shows significant increase. For *n* = 3 (close to the unconstrained angles), the dissociation energy has the expected Sn > Ge > Si order. Si and Ge show very similar binding energies for the different *n*’s, with an unfavorable binding for *n* = 3, but strong binding for *n* = 1 (D_e_ > 100 kJ mol^−1^). The difference in dissociation energies between **2_Si1_** and **2_Ge1_** is negligible, but α is smaller for Ge. This points at the same trend we saw for **1_T_**—the most dramatic increase in binding with smaller angles occurs with silicon (Figure S5). However, this does not actually make **2_Si1_** the stronger binder due to the smaller α in **2_Ge1_** (if both species had the same α angles, then **2_Si1_** would probably have the higher D_e_).

## 3. Conclusions

Besides the classical enhancement of the tetrel bond brought by having heavier tetrels, geometry can be an important factor in bond strength. In addition to the release of strain energy [17], a smaller angle between the substituents not only favors the bond by geometrically exposing the tetrel atom, but there is also an electronic effect that boosts the σ-hole and aids the charge transfer. These effects cannot appear in regular halogen bonding due to a lack of side substituents, but can be a feature in pnictogen and chalcogen bonds, or in hypercoordinated halogens [21]. Our findings suggest that, in designing new tetrel bonded complexes, focusing on the geometry around the tetrel atom could allow the use of the more abundant silicone compared to the heavier elements without significantly sacrificing binding strength.

## 4. Methods

All density functional theory (DFT) computations were done at the MN15/Def2-TZVPD [18,24] level with Gaussian16 [20]. All energies reported do not include ZPE correction. All minima were confirmed with frequency computations. In order to check the validity of the used DFT method, the dissociation energies for **1_Si_** and **1_Ge_** were computed with CCSD(T)/CBS (complete basis set extrapolation from aug-cc-pvtz/aug-cc-pvqz, carried out in ORCA [25]) at the geometries found by MN15/Def2-TZVPD (see Table S2). The CCSD(T)/CBS results showed values very close to those of DFT, with a maximum difference of 8.2 kJ mol^−1^, and displayed the same trends (stronger binding for **1_Ge_** at 109° and for **1_Si_** at 90°). NBO analyses were done with NBO3.1 [26] as appears in Gaussian16.

## Figures and Tables

**Table 1:** Properties of **1_T_** with different σ angles: σ_F-T_ and LUMO energies (kJ mol^−1^), F-T bond length (Å), %*s* and %*p* on T in the NBO F-T σ bond, and the maximal positive electrostatic potential at the σ hole (kJ mol^−1^).

T	α	σ_F-T_	LUMO	d_F-T_	%*s* ^b^	%*p* ^b^	V_s,max_
C	Opt. (109.1°) ^a^	−1413.9	154.7	1.376	21.52	78.28	81.2
	109°	−1413.2	154.7	1.376	21.48	78.32	81.2
	100°	−1328.6	147.6	1.426	16.09	83.71	84.1
	90°	−1232.6	84.6	1.511	8.53	91.25	95.2
Si	Opt. (108.3°) ^a^	−1328.8	27.2	1.598	21.40	76.03	142.5
	109°	−1332.2	28.7	1.596	21.62	75.81	137.3
	100°	−1282.5	−22.8	1.617	18.39	78.95	200.6
	90°	−1212.8	−123.7	1.655	13.54	83.79	256.5
Ge	Opt. (106.2°) ^a^	−1250.8	19.4	1.737	19.96	79.06	164.5
	109°	−1267.7	21.4	1.731	21.20	77.83	148.8
	100°	−1212.4	−18.6	1.751	17.05	81.98	197.0
	90°	−1145.6	−120.0	1.788	11.39	87.66	238.0
Sn	Opt. (104.4°) ^a^	−1158.6	−32.6	1.927	18.66	80.49	196.6
	109°	−1182.5	−46.3	1.920	20.75	78.41	170.9
	100°	−1134.9	−58.2	1.935	16.56	82.57	218.6
	90°	−1079.2	−158.9	1.962	11.20	87.94	255.7

^a^ Fully optimized molecule, with no angle restrictions. ^b^ %*s* and %*p* are the same for both the bonding and antibonding orbitals.

**Table 2:** Properties of the complexes of **1_T_** with HCN at different α angles: (distances in Å, energies in kJ mol^−1^).

T	α	d_T-F_	d_T···N_	% Cov. Rad. ^b^	D_e_^c^	*E* ^2^_*n*→σ*_^d^
C	Opt. (109.3°) ^a^	1.380	3.154	208	9.3	2.5
	109°	1.381	3.155	208	9.2	2.5
	100°	1.432	3.116	205	8.7	3.0
	90°	1.521	3.013	198	9.5	5.3
Si	Opt. (106.3°) ^a^	1.608	2.847	153	18.7	18.0
	109°	1.602	2.944	158	17.0	13.6
	100°	1.625	2.576	138	29.8	37.7
	90°	1.670	2.162	116	56.7	84.1 ^e^
Ge	Opt. (104.6°) ^a^	1.749	2.931	149	20.4	24.6
	109°	1.738	3.043	154	17.8	18.4
	100°	1.763	2.804	142	25.4	33.9
	90°	1.808	2.532	128	37.8	68.1
Sn	Opt. (102.2°) ^a^	1.945	2.934	136	25.7	27.0
	109°	1.930	3.086	143	20.8	17.4
	100°	1.950	2.887	134	28.9	31.0
	90°	1.982	2.703	125	39.4	54.3

^a^ Fully optimized molecule with no angle restrictions. ^b^ Ratio between tetrel bond and the sum of covalent radii of T and N. ^c^ Tetrel bond dissociation energies. ^d^ Perturbational stabilization energy according to NBO analysis corresponding to charge transfer. ^e^ *E*^2^_*n*→σ*_ for this complex was calculation by extrapolation, see Supplementary Information.

**Table 3:** Bond distances, angles, dissociation energies, and V_s.max_ of **2_T*n*_**, and their binding complexes with HCN (distances in Å, energies in kJ mol^−1^).

T	*n*	d_T-C_ Monomer	d_T-C_ Complex	α Monomer	α Complex	d_T···N_	% Cov. Rad. ^c^	D_e_	V_s,max_^d^
C	1	1.481	1.486	97.6	97.1	2.953	194.3	0.9	42.1
	2	1.561	-	107.8	-	-	-	NB	−107.2
	3	1.542	-	111.4	-	-	-	NB	−153.5
Si	1	1.884	1.954	88.7	83.8	1.931	103.8	111.3	260.3
	2	1.830	1.879	101.0	95.6	2.084	112.0	42.3	147.6
	3 ^a^	1.854	1.860	108.5	107.7	3.549	190.8	−3.2	−28.7
Ge	1	1.974	2.043	83.2	79.8	2.047	103.9	112.6	339.2
	2	1.899	1.935	98.9	95.4	2.220	112.7	47.9	186.6
	3	1.934	1.944	109.4	107.8	3.198	162.3	−1.2	9.8
Sn ^b^	2	2.077	2.104	93.0	91.0	2.318	107.3	73.9	264.5
	3	2.118	2.147	107.9	104.1	2.470	114.4	30.3	140.2

^a^ The complex with HCN interacting with the σ-hole is actually a maximum in energy as attraction between the oxygens and the positive charge of HCN are significant. ^b^ **2_Sn1_** is unstable. ^c^ Ratio of the tetrel bond and the sum of covalent radii of T and N. ^d^ Measured at the extension of the C-T bond.

## Supplementary Materials

The following are available online: Figures S1–S3: Chosen MOs and ESP maps for **1_C_**, **1_Ge_**, **1_Sn_** with three different F-T-H angles; Figure S4: graphs for the complexation of **1_T_** with HCN; Figure S5: graphs for the complexation of **2_T*n*_** with HCN.
