# Supplementary material for: On the Power of Geometry over Tetrel Bonds
Source: Molecules. 2018 Oct 24;23(11):2742. doi: 10.3390/molecules23112742 (PMC6278272; doi:10.3390/molecules23112742)
Supplement: Supplementary file 1 [file molecules-23-02742-s001.pdf]

# On the Power of the Geometry over the Tetrel Bond

**Ephrath Solel [1] and Sebastian Kozuch [1,*]**

[1] Department of Chemistry, Ben-Gurion University of the Negev, Beer-Sheva 841051, Israel

* Correspondence: kozuch@bgu.ac.il; Tel.: +972-8-64-61192

**$E^2_{n\to\sigma^*}$ for 1$_{Si}$**

$E^2_{n\to\sigma^*}$ was taken directly from the NBO analyses for all dimers in which the T···N interaction is noncovalent. According to NBO analyses the complex formed between 1$_{Si}$ at α=90° and HCN is covalent, so the $E^2_{n\to\sigma^*}$ value for it was computed by extrapolation. This was done by increasing the Si-N distance by steps of 0.1 Å (while keeping α constant) until the resulting complex was considered noncovalent in the NBO analyses. From four such complexes, and assuming a linear relationship between the $E^2_{n\to\sigma^*}$ and the Si-N distance, we extrapolated the value for the optimized Si-N distance.

**Frontier orbitals and ESP for 1$_T$:**

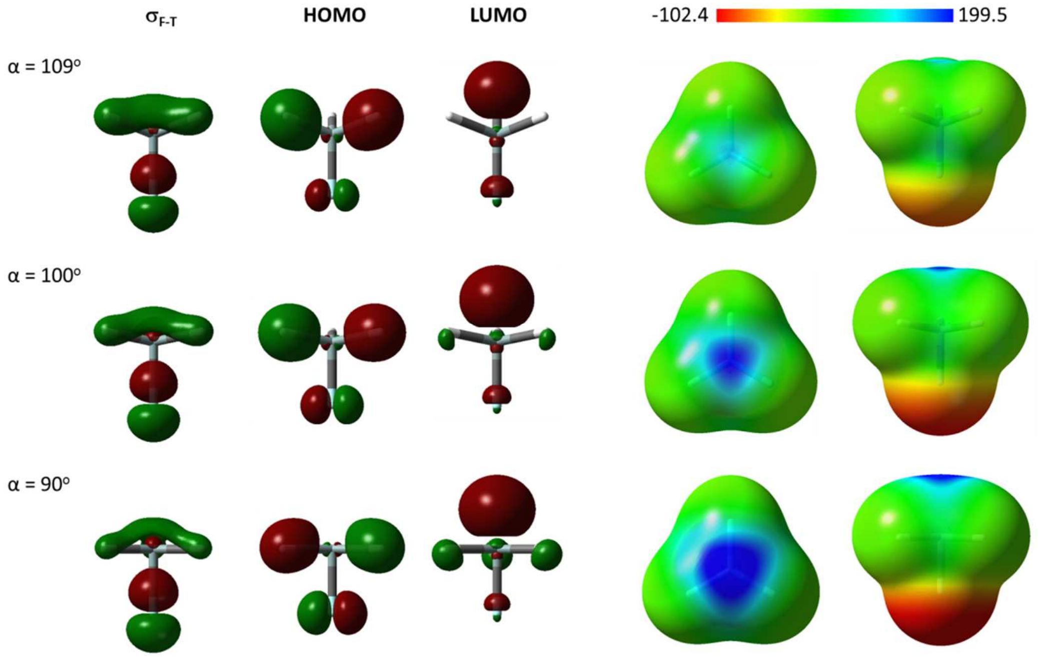


**Figure S1.** Chosen MOs and ESP maps for **1**$_{Si}$ with three different F-Si-H angles (α). The ESP maps are on the 0.001 density isosurface. The color scale is in kJ mol$^{-1}$. $\sigma_{F\text{-}T}$ corresponds to the bonding F-T σ orbital, irrespective of its position. The HOMO is doubly degenerate.

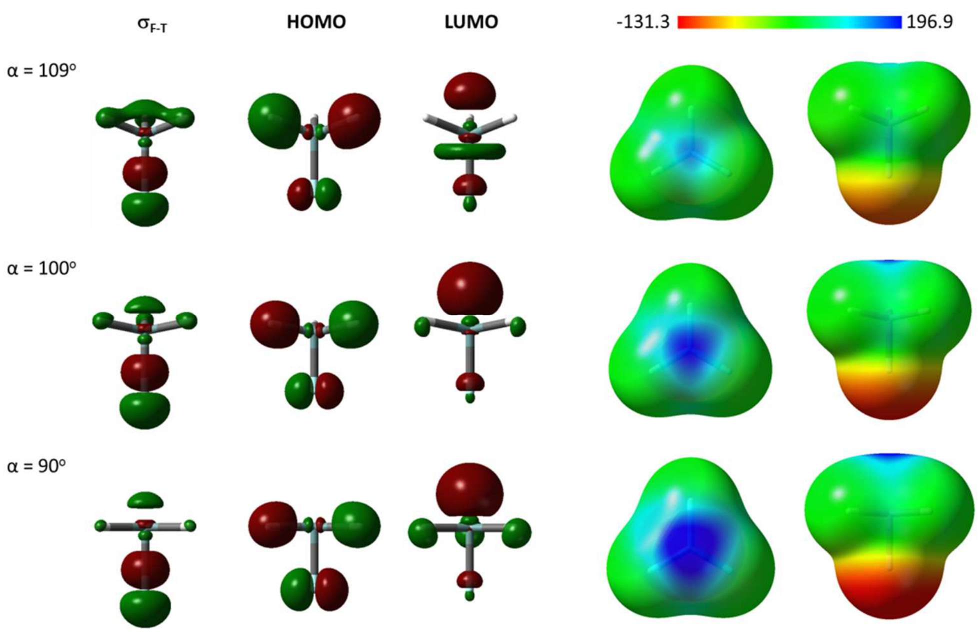


**Figure S2.** Chosen MOs and ESP maps for **1**$_{Ge}$ with three different F-Ge-H angles (α). The ESP maps are on the 0.001 density isosurface. The color scale is in kJ mol$^{-1}$. $\sigma_{F-T}$ corresponds to the bonding F-T σ orbital, irrespective of its position. The HOMO is doubly degenerate.

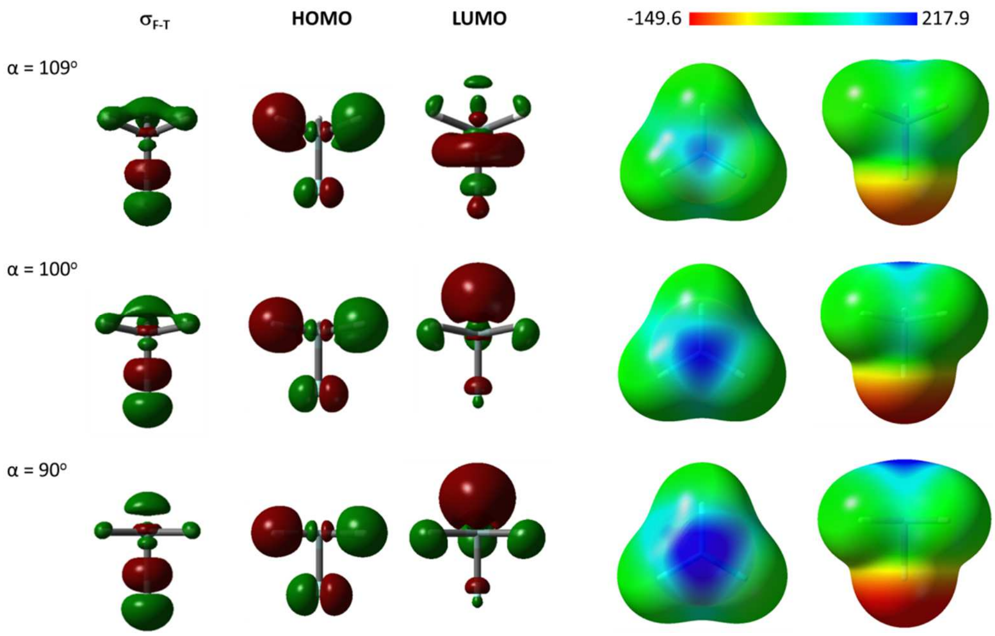


**Figure S3.** Chosen MOs and ESP maps for **1**$_{Sn}$ with three different F-Sn-H angles (α). The ESP maps are on the 0.001 density isosurface. The color scale is in kJ mol$^{-1}$. $\sigma_{F-T}$ corresponds to the bonding F-T σ orbital, irrespective of its position. The HOMO is doubly degenerate.

**Trends in 1T and 2Tn:**

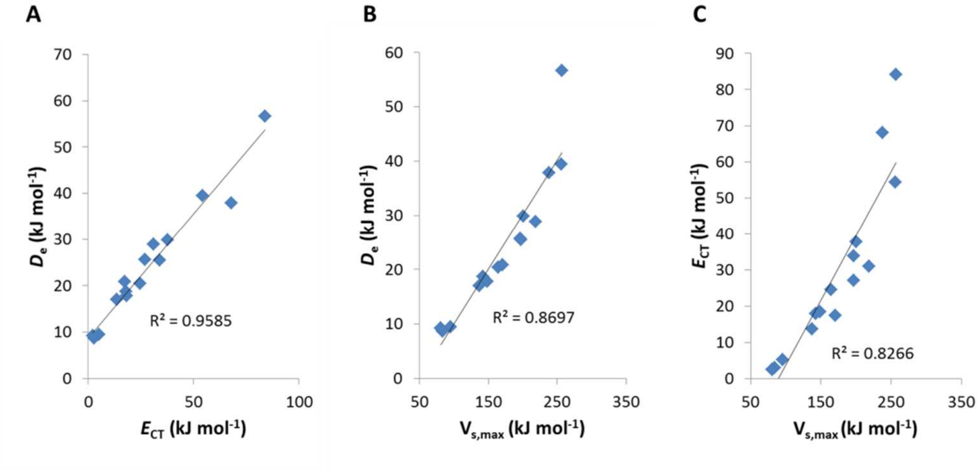


**Figure S4.** Complexation of **1T** with HCN: A) Dissociation energy as a function of the NBO $n \rightarrow \sigma^*$ charge transfer energy; B) Dissociation energy as a function of $V_{s,max}$; C) $n \rightarrow \sigma^*$ charge transfer energy as a function of of $V_{s,max}$.

**Table S1.** Distortion energies (in kJ mol$^{-1}$) relative to the unconstrained (optimized) reference structure, of the monomer and complex of **1T** with HCN at different α angles.

| T | α | $E_{dist}$ **monomer** | $E_{dist}$ **complex** | $\Delta E_{dist}$ |
|---|---|---|---|---|
| C | 109° | 0.0 | 0.0 | -0.0 |
| | 100° | 26.7 | 27.3 | -0.6 |
| | 90° | 108.5 | 108.3 | 0.2 |
| Si | 109° | 0.2 | 1.9 | -1.7 |
| | 100° | 21.0 | 9.9 | 11.1 |
| | 90° | 99.6 | 61.6 | 38.0 |
| Ge | 109° | 2.2 | 4.8 | -2.6 |
| | 100° | 10.2 | 5.2 | 5.0 |
| | 90° | 67.9 | 50.5 | 17.4 |
| Sn | 109° | 4.8 | 9.7 | -4.8 |
| | 100° | 4.3 | 1.1 | 3.2 |
| | 90° | 46.0 | 32.2 | 13.8 |

**Table S2.** Tetrel bond dissociation energies (in kJ mol$^{-1}$) for the complexes of **1T** (T = Si, Ge) with HCN at different α angles, computed at CCSD(T)/CBS//MN15/Def2-TZVPD, at MN15/Def2-TZVPD, and the difference between them.

| T | α | $D_e$[a] | $D_e$[b] | $D_{Diff}$[c] |
|---|---|---|---|---|
| Si | 109° | 13.6 | 17.0 | 3.3 |
| | 100° | 24.6 | 29.8 | 5.2 |
| | 90° | 48.5 | 56.7 | 8.2 |
| Ge | 109° | 17.5 | 17.8 | 0.3 |
| | 100° | 26.6 | 25.4 | 1.2 |
| | 90° | 41.2 | 37.8 | 3.4 |

[a] At CCSD(T)/CBS//MN15/Def2-TZVPD

[b] At MN15/Def2-TZVPD

[c] The absolute value of the difference in dissociation energies for the two methods

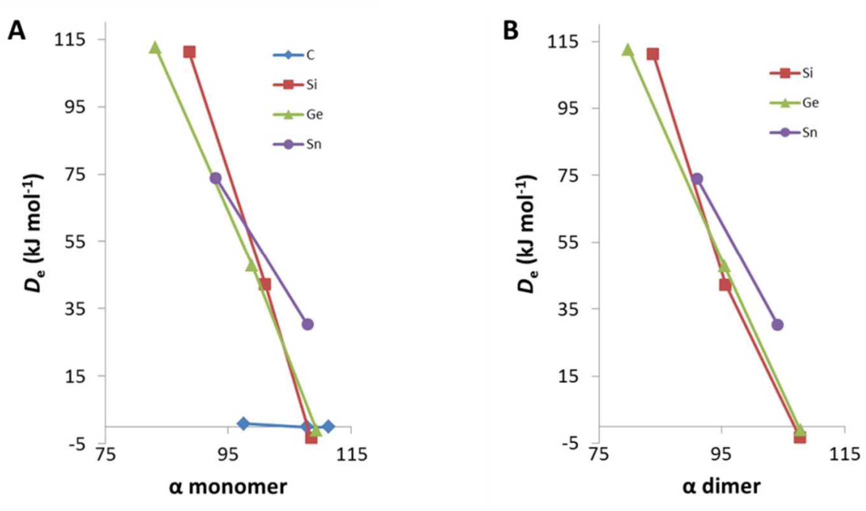


**Figure S5.** Complexation of **2**T*n* with HCN: dissociation energy as a function of the α angle in the A) monomer and B) dimer.

## Geometries:

**1**$_{C}$ $\alpha$=90°:

| | | | |
|---|---|---|---|
| C | 0.000000 | 0.000000 | -0.755472 |
| H | 0.000000 | 1.083039 | -0.755473 |
| H | 0.937939 | -0.541519 | -0.755473 |
| H | -0.937939 | -0.541519 | -0.755473 |
| F | 0.000000 | 0.000000 | 0.755472 |

**1**$_{C}$ $\alpha$=100°:

| | | | |
|---|---|---|---|
| C | 0.000000 | 0.000000 | -0.681405 |
| H | -0.000000 | 1.069603 | -0.870006 |
| H | 0.926304 | -0.534802 | -0.870006 |
| H | -0.926304 | -0.534802 | -0.870006 |
| F | 0.000000 | 0.000000 | 0.744272 |

**1**$_{C}$ $\alpha$=109°:

| | | | |
|---|---|---|---|
| C | 0.000000 | 0.000000 | -0.629039 |
| H | 0.000000 | 1.030579 | -0.983895 |
| H | 0.892508 | -0.515290 | -0.983895 |
| H | -0.892508 | -0.515290 | -0.983895 |
| F | 0.000000 | 0.000000 | 0.747324 |

**1**$_{C}$ fully optimized:

| | | | |
|---|---|---|---|
| C | 0.000000 | 0.000000 | -0.628620 |
| H | 0.000000 | 1.030265 | -0.984627 |
| H | 0.892235 | -0.515132 | -0.984627 |
| H | -0.892235 | -0.515132 | -0.984627 |
| F | 0.000000 | 0.000000 | 0.747289 |

**1**$_{C}$ ··· NCH $\alpha$=90°:

| | | | |
|---|---|---|---|
| C | 0.000000 | -0.000000 | 1.174332 |
| H | 0.000000 | 1.081632 | 1.174323 |
| H | 0.936720 | -0.540816 | 1.174323 |
| H | -0.936720 | -0.540816 | 1.174323 |
| F | 0.000000 | -0.000000 | 2.695395 |
| N | -0.000000 | 0.000000 | -1.838215 |
| C | -0.000000 | 0.000000 | -2.984634 |
| H | -0.000000 | 0.000000 | -4.052203 |

**1**$_{C}$ ··· NCH $\alpha$=100°:

| | | | |
|---|---|---|---|
| C | -0.000000 | -0.000000 | 1.262446 |
| H | 0.000000 | 1.068300 | 1.074068 |
| H | 0.925175 | -0.534150 | 1.074068 |
| H | -0.925175 | -0.534150 | 1.074068 |
| F | -0.000000 | -0.000000 | 2.694380 |
| N | 0.000000 | 0.000000 | -1.853786 |
| C | 0.000000 | 0.000000 | -3.000324 |
| H | 0.000000 | 0.000000 | -4.067853 |

**1**$_{C}$ ··· NCH $\alpha$=109°:

| | | | |
|---|---|---|---|
| C | 0.000000 | -0.000000 | 1.309149 |
| H | -0.000000 | 1.029740 | 0.954578 |
| H | 0.891781 | -0.514870 | 0.954578 |
| H | -0.891781 | -0.514870 | 0.954578 |
| F | 0.000000 | -0.000000 | 2.690398 |
| N | -0.000000 | 0.000000 | -1.845633 |
| C | -0.000000 | 0.000000 | -2.992178 |
| H | -0.000000 | 0.000000 | -4.059711 |

**1**$_{C}$ ··· NCH fully optimized:

| | | | |
|---|---|---|---|
| C | -0.000000 | 0.000000 | 1.309744 |
| H | 0.000000 | 1.028195 | 0.950218 |
| H | 0.890443 | -0.514098 | 0.950218 |
| H | -0.890443 | -0.514098 | 0.950218 |
| F | -0.000000 | 0.000000 | 2.689760 |
| N | 0.000000 | -0.000000 | -1.844545 |
| C | 0.000000 | -0.000000 | -2.991088 |
| H | 0.000000 | -0.000000 | -4.058621 |

**1**$_{Si}$ $\alpha$=90°:

| | | | |
|---|---|---|---|
| H | -0.000000 | 1.466232 | 0.572924 |
| H | -1.269794 | -0.733116 | 0.572924 |
| H | 1.269794 | -0.733116 | 0.572924 |
| F | 0.000000 | 0.000000 | -1.082190 |
| Si | 0.000000 | 0.000000 | 0.572924 |

**1**$_{Si}$ $\alpha$=100°:

| | | | |
|---|---|---|---|
| H | 0.000000 | 1.440434 | 0.784320 |
| H | -1.247452 | -0.720217 | 0.784320 |
| H | 1.247452 | -0.720217 | 0.784320 |
| F | 0.000000 | 0.000000 | -1.086402 |
| Si | 0.000000 | 0.000000 | 0.530333 |

**1**$_{Si}$ $\alpha$=109°:

| | | | |
|---|---|---|---|
| H | 0.000000 | 1.383161 | 0.973890 |
| H | -1.197852 | -0.691580 | 0.973890 |
| H | 1.197852 | -0.691580 | 0.973890 |
| F | 0.000000 | 0.000000 | -1.098721 |
| Si | 0.000000 | 0.000000 | 0.497630 |

**1**$_{Si}$ fully optimized:

| | | | |
|---|---|---|---|
| Si | 0.000000 | 0.000000 | 0.500026 |
| H | -0.000000 | 1.388888 | 0.959059 |
| H | -1.202813 | -0.694444 | 0.959059 |
| H | 1.202813 | -0.694444 | 0.959059 |
| F | 0.000000 | 0.000000 | -1.097505 |

**1**$_{Si}$ ··· NCH $\alpha$=90°:

| | | | |
|---|---|---|---|
| Si | -0.000000 | 0.000000 | 0.607414 |
| H | -0.000000 | 1.465849 | 0.607404 |
| H | 1.269463 | -0.732925 | 0.607404 |
| H | -1.269463 | -0.732925 | 0.607404 |
| F | -0.000000 | 0.000000 | 2.277133 |
| N | 0.000000 | -0.000000 | -1.554218 |
| C | 0.000000 | -0.000000 | -2.696003 |
| H | 0.000000 | -0.000000 | -3.764671 |

**1**$_{Si}$ ··· NCH $\alpha$=100°:

| | | | |
|---|---|---|---|
| Si | 0.000000 | -0.000000 | 0.781847 |
| H | 0.000000 | 1.439174 | 0.528073 |
| H | 1.246361 | -0.719587 | 0.528073 |
| H | -1.246361 | -0.719587 | 0.528073 |
| F | 0.000000 | -0.000000 | 2.407192 |
| N | -0.000000 | 0.000000 | -1.794049 |
| C | -0.000000 | 0.000000 | -2.938358 |
| H | -0.000000 | 0.000000 | -4.006316 |

**1**$_{Si}$ ··· NCH $\alpha$=109°:

| | | | |
|---|---|---|---|
| Si | -0.000000 | -0.000000 | 0.932964 |
| H | -0.000000 | 1.382198 | 0.457046 |
| H | 1.197018 | -0.691099 | 0.457046 |
| H | -1.197018 | -0.691099 | 0.457046 |
| F | -0.000000 | -0.000000 | 2.535132 |
| N | 0.000000 | 0.000000 | -2.011491 |
| C | 0.000000 | 0.000000 | -3.157257 |
| H | 0.000000 | 0.000000 | -4.224836 |

**1**$_{Si}$ ··· NCH fully optimized:

| | | | |
|---|---|---|---|
| Si | -0.000000 | 0.000000 | 0.892493 |
| H | -0.000000 | 1.402275 | 0.481864 |
| H | 1.214406 | -0.701138 | 0.481864 |
| H | -1.214406 | -0.701138 | 0.481864 |
| F | -0.000000 | 0.000000 | 2.500362 |
| N | 0.000000 | -0.000000 | -1.954139 |
| C | 0.000000 | -0.000000 | -3.099589 |
| H | 0.000000 | -0.000000 | -4.167250 |

**1**$_{Ge}$ $\alpha$=90°:

| | | | |
|---|---|---|---|
| H | 0.000000 | 1.519678 | 0.365633 |
| H | -1.316080 | -0.759839 | 0.365633 |
| H | 1.316080 | -0.759839 | 0.365633 |
| F | 0.000000 | 0.000000 | -1.421905 |
| Ge | 0.000000 | 0.000000 | 0.365633 |

**1**$_{Ge}$ $\alpha$=100°:

| | | | |
|---|---|---|---|
| H | -0.000000 | 1.496210 | 0.604012 |
| H | -1.295756 | -0.748105 | 0.604012 |
| H | 1.295756 | -0.748105 | 0.604012 |
| F | 0.000000 | 0.000000 | -1.410901 |
| Ge | 0.000000 | 0.000000 | 0.340190 |

**1**$_{Ge}$ $\alpha$=109°:

| | | | |
|---|---|---|---|
| H | 0.000000 | 1.438734 | 0.815650 |
| H | -1.245980 | -0.719367 | 0.815650 |
| H | 1.245980 | -0.719367 | 0.815650 |
| F | 0.000000 | 0.000000 | -1.410564 |
| Ge | 0.000000 | 0.000000 | 0.320254 |

**1**$_{Ge}$ fully optimized:

| | | | |
|---|---|---|---|
| H | 0.000000 | 1.460188 | 0.749689 |
| H | -1.264560 | -0.730094 | 0.749689 |
| H | 1.264560 | -0.730094 | 0.749689 |
| F | 0.000000 | 0.000000 | -1.410193 |
| Ge | 0.000000 | 0.000000 | 0.326333 |

**1**$_{Ge}$ ··· NCH $\alpha$=90°:

| | | | |
|---|---|---|---|
| Ge | 0.000000 | -0.000000 | 0.487146 |
| H | 0.000000 | 1.517418 | 0.487137 |
| H | 1.314123 | -0.758709 | 0.487137 |

| | | | |
|---|---|---|---|
| H | -1.314123 | -0.758709 | 0.487137 |
| F | 0.000000 | -0.000000 | 2.295085 |
| N | -0.000000 | 0.000000 | -2.045136 |
| C | -0.000000 | 0.000000 | -3.188781 |
| H | -0.000000 | 0.000000 | -4.257209 |

$\mathbf{1}_{Ge}$ ⋯ NCH $\alpha$=100°:

| | | | |
|---|---|---|---|
| Ge | -0.000000 | -0.000000 | 0.573495 |
| H | 0.000000 | 1.494285 | 0.310004 |
| H | 1.294088 | -0.747142 | 0.310004 |
| H | -1.294088 | -0.747142 | 0.310004 |
| F | -0.000000 | -0.000000 | 2.336852 |
| N | 0.000000 | 0.000000 | -2.230746 |
| C | 0.000000 | 0.000000 | -3.375766 |
| H | 0.000000 | 0.000000 | -4.443694 |

$\mathbf{1}_{Ge}$ ⋯ NCH $\alpha$=109°:

| | | | |
|---|---|---|---|
| Ge | -0.000000 | 0.000000 | 0.646989 |
| H | 0.000000 | 1.437976 | 0.151840 |
| H | 1.245324 | -0.718988 | 0.151840 |
| H | -1.245324 | -0.718988 | 0.151840 |
| F | -0.000000 | 0.000000 | 2.385213 |
| N | 0.000000 | -0.000000 | -2.395541 |
| C | 0.000000 | -0.000000 | -3.541378 |
| H | 0.000000 | -0.000000 | -4.609024 |

$\mathbf{1}_{Ge}$ ⋯ NCH fully optimized:

| | | | |
|---|---|---|---|
| Ge | 0.000000 | -0.000000 | 0.612460 |
| H | 0.000000 | 1.469487 | 0.229190 |
| H | 1.272613 | -0.734744 | 0.229190 |
| H | -1.272613 | -0.734744 | 0.229190 |
| F | 0.000000 | -0.000000 | 2.361845 |
| N | -0.000000 | 0.000000 | -2.318334 |
| C | -0.000000 | 0.000000 | -3.463826 |
| H | -0.000000 | 0.000000 | -4.531601 |

$\mathbf{1}_{Sn}$ $\alpha$=90°:

| | | | |
|---|---|---|---|
| H | -0.000000 | 1.686842 | 0.284764 |
| H | -1.460848 | -0.843421 | 0.284764 |
| H | 1.460848 | -0.843421 | 0.284764 |
| F | 0.000000 | 0.000000 | -1.676946 |
| Sn | 0.000000 | 0.000000 | 0.284764 |

$\mathbf{1}_{Sn}$ $\alpha$=100°:

| | | | |
|---|---|---|---|
| H | 0.000000 | 1.662037 | 0.559796 |
| H | -1.439366 | -0.831018 | 0.559796 |
| H | 1.439366 | -0.831018 | 0.559796 |
| F | 0.000000 | 0.000000 | -1.668453 |
| Sn | 0.000000 | 0.000000 | 0.266734 |

$\mathbf{1}_{Sn}$ $\alpha$=109°:

| | | | |
|---|---|---|---|
| H | -0.000000 | 1.600642 | 0.803116 |
| H | -1.386196 | -0.800321 | 0.803116 |
| H | 1.386196 | -0.800321 | 0.803116 |
| F | 0.000000 | 0.000000 | -1.667539 |
| Sn | 0.000000 | 0.000000 | 0.251970 |

$\mathbf{1}_{Sn}$ fully optimized:

| | | | |
|---|---|---|---|
| H | -0.000000 | 1.636704 | 0.679241 |
| H | -1.417427 | -0.818352 | 0.679241 |
| H | 1.417427 | -0.818352 | 0.679241 |
| F | 0.000000 | 0.000000 | -1.667476 |
| Sn | 0.000000 | 0.000000 | 0.259391 |

$\mathbf{1}_{Sn}$ ⋯ NCH $\alpha$=90°:

| | | | |
|---|---|---|---|
| Sn | -0.000000 | 0.000000 | 0.382533 |
| H | 0.000000 | 1.686241 | 0.382524 |
| H | 1.460328 | -0.843121 | 0.382524 |
| H | -1.460328 | -0.843121 | 0.382524 |
| F | -0.000000 | 0.000000 | 2.364746 |
| N | 0.000000 | 0.000000 | -2.320115 |
| C | 0.000000 | 0.000000 | -3.463937 |
| H | 0.000000 | 0.000000 | -4.532509 |

$\mathbf{1}_{Sn}$ ⋯ NCH $\alpha$=100°:

| | | | |
|---|---|---|---|
| Sn | 0.000000 | 0.000000 | 0.431890 |
| H | -0.000000 | 1.661370 | 0.138941 |
| H | 1.438789 | -0.830685 | 0.138941 |
| H | -1.438789 | -0.830685 | 0.138941 |
| F | 0.000000 | 0.000000 | 2.382010 |
| N | -0.000000 | -0.000000 | -2.454840 |
| C | -0.000000 | -0.000000 | -3.599632 |
| H | -0.000000 | -0.000000 | -4.667715 |

$\mathbf{1}_{Sn}$ ⋯ NCH $\alpha$=109°:

| | | | |
|---|---|---|---|
| Sn | -0.000000 | -0.000000 | 0.481190 |
| H | 0.000000 | 1.599771 | -0.069659 |
| H | 1.385442 | -0.799886 | -0.069659 |
| H | -1.385442 | -0.799886 | -0.069659 |
| F | -0.000000 | -0.000000 | 2.411590 |
| N | 0.000000 | 0.000000 | -2.604857 |
| C | 0.000000 | 0.000000 | -3.750442 |
| H | 0.000000 | 0.000000 | -4.818200 |

$\mathbf{1}_{Sn}$ ⋯ NCH fully optimized:

| | | | |
|---|---|---|---|
| Sn | -0.000000 | -0.000000 | 0.443836 |
| H | 0.000000 | 1.649484 | 0.086319 |
| H | 1.428495 | -0.824742 | 0.086319 |
| H | -1.428495 | -0.824742 | 0.086319 |
| F | -0.000000 | -0.000000 | 2.388409 |
| N | 0.000000 | 0.000000 | -2.490243 |
| C | 0.000000 | 0.000000 | -3.635250 |
| H | 0.000000 | 0.000000 | -4.703241 |

$\mathbf{2}_{C1}$ :

| | | | |
|---|---|---|---|
| C | 0.000000 | 0.000000 | 0.619422 |
| C | 0.000000 | 0.000000 | -0.861707 |
| C | -1.318537 | 0.761025 | -0.656471 |
| H | -1.318698 | 1.795903 | -0.999146 |
| H | -2.214727 | 0.243369 | -0.998929 |
| C | 0.000201 | -1.522399 | -0.656471 |
| C | 1.318335 | 0.761374 | -0.656471 |
| H | 0.896600 | -2.039694 | -0.998929 |
| H | -0.895948 | -2.039977 | -0.999146 |
| H | 1.318127 | 1.796325 | -0.998929 |
| H | 2.214646 | 0.244075 | -0.999146 |
| O | -1.196400 | 0.690742 | 0.802684 |
| O | -0.000000 | -1.381483 | 0.802684 |
| O | 1.196400 | 0.690742 | 0.802684 |

$\mathbf{2}_{C2}$ :

| | | | |
|---|---|---|---|
| C | 0.000000 | 0.000000 | 0.730197 |
| C | 0.000000 | 0.000000 | -0.830641 |
| C | 1.477389 | 1.582397 | 0.129294 |
| C | -2.109090 | 0.488258 | 0.129294 |
| C | 0.631701 | -2.070654 | 0.129294 |
| C | -1.314549 | 0.707676 | -1.154892 |
| H | -2.913834 | 1.202018 | 0.292148 |
| H | -2.519097 | -0.528600 | 0.168937 |
| H | -1.143376 | 1.778688 | -1.296150 |
| H | -1.806131 | 0.311760 | -2.044461 |
| C | 0.044409 | -1.492271 | -1.154892 |
| C | 1.270140 | 0.784595 | -1.154892 |
| H | 1.717329 | -1.917303 | 0.168937 |
| H | 0.415939 | -3.124463 | 0.292148 |
| H | 0.633073 | -1.720036 | -2.044461 |
| H | -0.968701 | -1.879536 | -1.296150 |
| H | 2.497895 | 1.922445 | 0.292148 |
| H | 0.801768 | 2.445902 | 0.168937 |
| H | 1.173058 | 1.408275 | -2.044461 |
| H | 2.112077 | 0.100849 | -1.296150 |
| O | 1.142373 | 0.659549 | 1.154251 |
| O | -1.142373 | 0.659549 | 1.154251 |
| O | 0.000000 | -1.319098 | 1.154251 |

$\mathbf{2}_{C3}$ :

| | | | |
|---|---|---|---|
| C | 0.000000 | 0.000000 | 0.720326 |
| C | 0.000000 | 0.000000 | -0.822103 |
| C | 2.361066 | -0.070681 | 0.849087 |
| C | -1.119321 | 2.080084 | 0.849087 |
| C | -1.241745 | -2.009403 | 0.849087 |
| C | -1.140513 | 0.896301 | -1.322019 |
| H | -1.011043 | 3.027776 | 1.375103 |
| H | -2.037397 | 1.593395 | 1.193683 |
| C | -0.205963 | -1.435864 | -1.322019 |
| C | 1.346476 | 0.539563 | -1.322019 |
| H | -0.361222 | -2.561135 | 1.193683 |
| H | -2.116610 | -2.389477 | 1.375103 |
| H | 3.127652 | -0.638299 | 1.375103 |
| H | 2.398619 | 0.967740 | 1.193683 |

| | | | |
|---|---|---|---|
| C | 2.537321 | -0.137662 | -0.655001 |
| C | -1.387879 | -2.128554 | -0.655001 |
| C | -1.149442 | 2.266215 | -0.655001 |
| H | -2.335307 | -1.681053 | -0.964525 |
| H | -1.421845 | -3.182765 | -0.939718 |
| H | 2.623488 | -1.181909 | -0.964525 |
| H | 3.467278 | 0.360028 | -0.939718 |
| H | -2.045432 | 2.822736 | -0.939718 |
| H | -0.288181 | 2.862962 | -0.964525 |
| H | 0.693839 | -2.020445 | -1.111294 |
| H | -0.325428 | -1.406615 | -2.410831 |
| H | 1.380879 | 0.421479 | -2.410831 |
| H | 1.402837 | 1.611105 | -1.111294 |
| H | -2.096676 | 0.409340 | -1.111294 |
| H | -1.055451 | 0.985137 | -2.410831 |
| O | 0.000000 | 1.294336 | 1.228592 |
| O | -1.120928 | -0.647168 | 1.228592 |
| O | 1.120928 | -0.647168 | 1.228592 |

$\mathbf{2}_{C1}$ ··· NCH :

| | | | |
|---|---|---|---|
| C | 0.000000 | 0.000000 | -0.172349 |
| C | 0.000000 | 0.000000 | -1.658067 |
| C | -1.415952 | -0.555519 | -1.456447 |
| H | -2.224031 | 0.090197 | -1.802663 |
| H | -1.570138 | -1.577926 | -1.803839 |
| C | 1.189069 | -0.948491 | -1.456447 |
| C | 0.226882 | 1.504010 | -1.456447 |
| H | 2.151593 | -0.570816 | -1.803839 |
| H | 1.033903 | -1.971166 | -1.802663 |
| H | -0.581455 | 2.148743 | -1.803839 |
| H | 1.190129 | 1.880969 | -1.802663 |
| N | 0.000000 | 0.000000 | 2.781072 |
| C | 0.000000 | 0.000000 | 3.927526 |
| H | 0.000000 | 0.000000 | 4.994341 |
| O | -1.288921 | -0.506723 | -0.000373 |
| O | 1.083295 | -0.862877 | -0.000373 |
| O | 0.205626 | 1.369600 | -0.000373 |

$\mathbf{2}_{Si1}$ :

| | | | |
|---|---|---|---|
| Si | 0.000000 | 0.000000 | 0.767793 |
| C | 0.000000 | 0.000000 | -1.116337 |
| C | 0.000061 | 1.476967 | -0.815534 |
| H | 0.898427 | 1.989664 | -1.150611 |
| H | -0.898214 | 1.989755 | -1.150725 |
| C | -1.279122 | -0.738431 | -0.815534 |
| C | 1.279061 | -0.738536 | -0.815534 |
| H | -1.274071 | -1.772753 | -1.150725 |
| H | -2.172313 | -0.216771 | -1.150611 |
| H | 2.172285 | -0.217001 | -1.150725 |
| H | 1.273886 | -1.772893 | -1.150611 |
| O | 0.000000 | 1.609000 | 0.730523 |
| O | -1.393435 | -0.804500 | 0.730523 |
| O | 1.393435 | -0.804500 | 0.730523 |

$\mathbf{2}_{Si2}$ :

| | | | |
|---|---|---|---|
| Si | 0.000000 | 0.000000 | 0.856306 |
| C | 0.000000 | 0.000000 | -0.974109 |
| C | 0.000000 | 2.303015 | -0.046204 |
| C | -1.994469 | -1.151507 | -0.046204 |
| C | 1.994469 | -1.151507 | -0.046204 |
| C | -1.470025 | -0.344776 | -1.251546 |
| H | -3.082753 | -1.169763 | -0.000237 |
| H | -1.636467 | -2.186223 | -0.097962 |
| H | -2.045988 | 0.584679 | -1.319537 |
| H | -1.628861 | -0.898551 | -2.182162 |
| C | 1.033597 | -1.100691 | -1.251546 |
| C | 0.436428 | 1.445467 | -1.251546 |
| H | 2.711558 | -0.324110 | -0.097962 |
| H | 2.554421 | -2.084861 | -0.000237 |
| H | 1.592599 | -0.961360 | -2.182162 |
| H | 0.516647 | -2.064217 | -1.319537 |
| H | 0.528332 | 3.254624 | -0.000237 |
| H | -1.075091 | 2.510333 | -0.097962 |
| H | 0.036262 | 1.859911 | -2.182162 |
| H | 1.529341 | 1.479538 | -1.319537 |
| O | 0.275997 | 1.580770 | 1.167315 |
| O | -1.506985 | -0.551364 | 1.167315 |
| O | 1.230988 | -1.029406 | 1.167315 |

$\mathbf{2}_{Si3}$ :

| | | | |
|---|---|---|---|
| Si | 0.000000 | 0.000000 | 0.878365 |
| C | 0.000000 | 0.000000 | -0.975652 |
| C | 0.000000 | 2.615475 | 0.709908 |
| C | -2.265067 | -1.307737 | 0.709908 |
| C | 2.265067 | -1.307737 | 0.709908 |
| C | -1.028949 | -1.026834 | -1.485337 |
| H | -3.250529 | -1.347590 | 1.175691 |
| H | -1.790173 | -2.288474 | 0.845396 |
| C | 1.403738 | -0.377679 | -1.485337 |
| C | -0.374790 | 1.404512 | -1.485337 |
| H | 2.876963 | -0.406098 | 0.845396 |
| H | 2.792312 | -2.141245 | 1.175691 |
| H | 0.458217 | 3.488836 | 1.175691 |
| H | -1.086790 | 2.694572 | 0.845396 |
| C | 0.334281 | 2.556753 | -0.772495 |
| C | 2.047073 | -1.567872 | -0.772495 |
| C | -2.381354 | -0.988881 | -0.772495 |
| H | 1.429947 | -2.465533 | -0.877210 |
| H | 3.012990 | -1.792775 | -1.232759 |
| H | 1.420241 | 2.471137 | -0.877210 |
| H | 0.046094 | 3.505713 | -1.232759 |
| H | -3.059083 | -1.712938 | -1.232759 |
| H | -2.850188 | -0.005604 | -0.877210 |
| H | 2.074087 | 0.482240 | -1.370132 |
| H | 1.335830 | -0.574067 | -2.564756 |
| H | -0.170759 | 1.443896 | -2.564756 |
| H | -1.454676 | 1.555092 | -1.370132 |
| H | -0.619411 | -2.037333 | -1.370132 |
| H | -1.165071 | -0.869829 | -2.564756 |
| O | -1.510726 | -0.315984 | 1.395447 |
| O | 1.029013 | -1.150335 | 1.395447 |
| O | 0.481713 | 1.466319 | 1.395447 |

$\mathbf{2}_{Si1}$ ··· NCH :

| | | | |
|---|---|---|---|
| Si | 0.000000 | 0.000000 | 0.272760 |
| C | 0.000000 | 0.000000 | -1.681111 |
| C | -1.375466 | -0.539666 | -1.405430 |
| H | -2.173122 | 0.107698 | -1.771496 |
| H | -1.519939 | -1.556877 | -1.771135 |
| C | 1.155098 | -0.921356 | -1.405430 |
| C | 0.220368 | 1.461022 | -1.405430 |
| H | 2.108265 | -0.537868 | -1.771135 |
| H | 0.993292 | -1.935828 | -1.771496 |
| H | -0.588325 | 2.094745 | -1.771135 |
| H | 1.179831 | 1.828130 | -1.771496 |
| N | 0.000000 | 0.000000 | 2.204119 |
| C | 0.000000 | 0.000000 | 3.341147 |
| H | 0.000000 | 0.000000 | 4.410676 |
| O | -1.513413 | -0.593449 | 0.096136 |
| O | 1.270649 | -1.013930 | 0.096136 |
| O | 0.242764 | 1.607379 | 0.096136 |

$\mathbf{2}_{Si2}$ ··· NCH :

| | | | |
|---|---|---|---|
| Si | 0.000000 | 0.000000 | 0.484693 |
| C | 0.000000 | 0.000000 | -1.394714 |
| C | 0.457164 | 2.278782 | -0.596403 |
| C | -2.202065 | -0.743476 | -0.596403 |
| C | 1.744901 | -1.535306 | -0.596403 |
| C | -1.490559 | 0.015518 | -1.724558 |
| H | -3.266697 | -0.508139 | -0.548318 |
| H | -2.106226 | -1.825482 | -0.749405 |
| H | -1.847951 | 1.051934 | -1.725092 |
| H | -1.738397 | -0.415363 | -2.701453 |
| C | 0.731840 | -1.298621 | -1.724558 |
| C | 0.758718 | 1.283102 | -1.724558 |
| H | 2.634027 | -0.911304 | -0.749405 |
| H | 2.073410 | -2.574974 | -0.548318 |
| H | 1.228913 | -1.297814 | -2.701453 |
| H | 0.012974 | -2.126339 | -1.725092 |
| H | 1.193288 | 3.083112 | -0.548318 |
| H | -0.527801 | 2.736786 | -0.749405 |
| H | 0.509484 | 1.713177 | -2.701453 |
| H | 1.834977 | 1.074405 | -1.725092 |
| N | 0.000000 | 0.000000 | 2.568779 |
| C | 0.000000 | 0.000000 | 3.707518 |
| H | 0.000000 | 0.000000 | 4.775091 |

| | | | |
|---|---|---|---|
| O | −1.603528 | −0.397832 | 0.647127 |
| O | 0.457231 | 1.587612 | 0.647127 |
| O | 1.146297 | −1.189780 | 0.647127 |

$\mathbf{2}_{Si3}$ ··· NCH :

| | | | |
|---|---|---|---|
| Si | 0.000000 | 0.000000 | 0.344652 |
| C | 0.000000 | 0.000000 | −1.514954 |
| C | 2.612037 | 0.112482 | 0.159181 |
| C | −1.403431 | 2.205850 | 0.159181 |
| C | −1.208607 | −2.318331 | 0.159181 |
| C | −1.077748 | 0.972740 | −2.028511 |
| H | −1.488436 | 3.191009 | 0.620385 |
| H | −2.359291 | 1.684868 | 0.304734 |
| C | −0.303543 | −1.419727 | −2.028511 |
| C | 1.381292 | 0.446987 | −2.028511 |
| H | −0.279493 | −2.885640 | 0.304734 |
| H | −2.019277 | −2.884528 | 0.620385 |
| H | 3.507713 | −0.306481 | 0.620385 |
| H | 2.638784 | 1.200772 | 0.304734 |
| C | 2.570578 | −0.208901 | −1.326579 |
| C | −1.466203 | −2.121736 | −1.326579 |
| C | −1.104375 | 2.330637 | −1.326579 |
| H | −2.393778 | −1.552891 | −1.443001 |
| H | −1.635243 | −3.100187 | −1.784780 |
| H | 2.541732 | −1.296627 | −1.443001 |
| H | 3.502462 | 0.133932 | −1.784780 |
| H | −1.867219 | 2.966255 | −1.784780 |
| H | −0.147954 | 2.849518 | −1.443001 |
| H | 0.587005 | −2.047289 | −1.904838 |
| H | −0.492920 | −1.361409 | −3.110170 |
| H | 1.425475 | 0.253823 | −3.110170 |
| H | 1.479502 | 1.532006 | −1.904838 |
| H | −2.066507 | 0.515283 | −1.904838 |
| H | −0.932555 | 1.107586 | −3.110170 |
| N | 0.000000 | 0.000000 | 3.893924 |
| C | 0.000000 | 0.000000 | 5.040992 |
| H | 0.000000 | 0.000000 | 6.107870 |
| O | −1.118107 | −1.074880 | 0.838861 |
| O | 1.489927 | −0.430870 | 0.838861 |
| O | −0.371819 | 1.505749 | 0.838861 |

$\mathbf{2}_{Ge1}$ :

| | | | |
|---|---|---|---|
| Ge | 0.000000 | 0.000000 | 0.704037 |
| C | 0.000000 | 0.000000 | −1.269894 |
| C | 0.000000 | 1.489802 | −1.025291 |
| H | 0.896349 | 1.984103 | −1.396516 |
| H | −0.896554 | 1.983901 | −1.396287 |
| C | −1.290207 | −0.744901 | −1.025291 |
| C | 1.290207 | −0.744901 | −1.025291 |
| H | −1.269831 | −1.768389 | −1.396287 |
| H | −2.166458 | −0.215791 | −1.396516 |
| H | 2.166385 | −0.215512 | −1.396287 |
| H | 1.270109 | −1.768312 | −1.396516 |
| O | 0.000134 | 1.738274 | 0.496826 |
| O | −1.505457 | −0.869021 | 0.496826 |
| O | 1.505323 | −0.869253 | 0.496826 |

$\mathbf{2}_{Ge2}$ :

| | | | |
|---|---|---|---|
| Ge | 0.000000 | 0.000000 | 0.760937 |
| C | 0.000000 | 0.000000 | −1.138248 |
| C | 0.000000 | 2.337331 | −0.226642 |
| C | −2.024188 | −1.168665 | −0.226642 |
| C | 2.024188 | −1.168665 | −0.226642 |
| C | −1.474139 | −0.333745 | −1.404295 |
| H | −3.113164 | −1.214005 | −0.244661 |
| H | −1.640224 | −2.194596 | −0.289195 |
| H | −2.043709 | 0.600744 | −1.455980 |
| H | −1.636134 | −0.868651 | −2.345613 |
| C | 1.026101 | −1.109769 | −1.404295 |
| C | 0.448038 | 1.443514 | −1.404295 |
| H | 2.720688 | −0.323178 | −0.289195 |
| H | 2.607942 | −2.089077 | −0.244661 |
| H | 1.570341 | −0.982608 | −2.345613 |
| H | 0.501595 | −2.070276 | −1.455980 |
| H | 0.505223 | 3.303082 | −0.244661 |
| H | −1.080464 | 2.517774 | −0.289195 |
| H | 0.065793 | 1.851259 | −2.345613 |
| H | 1.542115 | 1.469532 | −1.455980 |
| O | 0.300006 | 1.716326 | 1.035113 |
| O | −1.636385 | −0.598350 | 1.035113 |
| O | 1.336379 | −1.117976 | 1.035113 |

$\mathbf{2}_{Ge3}$ :

| | | | |
|---|---|---|---|
| Ge | 0.000000 | 0.000000 | 0.778694 |
| C | 0.000000 | 0.000000 | −1.154823 |
| C | 0.000000 | 2.672026 | 0.560909 |
| C | −2.314042 | −1.336013 | 0.560909 |
| C | 2.314042 | −1.336013 | 0.560909 |
| C | −1.079389 | −0.978149 | −1.645316 |
| H | −3.312350 | −1.431633 | 0.991746 |
| H | −1.832687 | −2.322834 | 0.621843 |
| C | 1.386797 | −0.445704 | −1.645316 |
| C | −0.307408 | 1.423853 | −1.645316 |
| H | 2.927977 | −0.425736 | 0.621843 |
| H | 2.896006 | −2.152763 | 0.991746 |
| H | 0.416344 | 3.584396 | 0.991746 |
| H | −1.095290 | 2.748570 | 0.621843 |
| C | 0.421146 | 2.543121 | −0.898406 |
| C | 1.991834 | −1.636284 | −0.898406 |
| C | −2.412980 | −0.906837 | −0.898406 |
| H | 1.318736 | −2.499639 | −0.928506 |
| H | 2.915466 | −1.939918 | −1.398548 |
| H | 1.505383 | 2.391878 | −0.928506 |
| H | 0.222285 | 3.494826 | −1.398548 |
| H | −3.137751 | −1.554908 | −1.398548 |
| H | −2.824119 | 0.107761 | −0.928506 |
| H | 2.087228 | 0.393692 | −1.562981 |
| H | 1.303709 | −0.673085 | −2.717436 |
| H | −0.068946 | 1.465587 | −2.717436 |
| H | −1.384561 | 1.610747 | −1.562981 |
| H | −0.702667 | −2.004439 | −1.562981 |
| H | −1.234763 | −0.792503 | −2.717436 |
| O | −1.607055 | −0.398860 | 1.361793 |
| O | 1.148951 | −1.192320 | 1.361793 |
| O | 0.458104 | 1.591181 | 1.361793 |

$\mathbf{2}_{Ge1}$ ··· NCH :

| | | | |
|---|---|---|---|
| Ge | 0.000000 | 0.000000 | 0.277302 |
| C | 0.000000 | 0.000000 | −1.766070 |
| C | −0.542027 | 1.381508 | −1.530223 |
| H | 0.109794 | 2.165557 | −1.918845 |
| H | −1.552925 | 1.513357 | −1.919239 |
| C | −0.925408 | −1.160164 | −1.530223 |
| C | 1.467435 | −0.221345 | −1.530223 |
| H | −0.534143 | −2.101551 | −1.919239 |
| H | −1.930324 | −0.987694 | −1.918845 |
| H | 2.087068 | 0.588194 | −1.919239 |
| H | 1.820530 | −1.177862 | −1.918845 |
| N | 0.000000 | 0.000000 | 2.324030 |
| C | 0.000000 | 0.000000 | 3.461996 |
| H | 0.000000 | 0.000000 | 4.532222 |
| O | −0.632945 | 1.612327 | −0.032973 |
| O | −1.079843 | −1.354310 | −0.032973 |
| O | 1.712788 | −0.258017 | −0.032973 |

$\mathbf{2}_{Ge2}$ ··· NCH :

| | | | |
|---|---|---|---|
| Ge | 0.000000 | 0.000000 | 0.412201 |
| C | 0.000000 | 0.000000 | −1.522362 |
| C | 0.475137 | 2.305683 | −0.696467 |
| C | −2.234348 | −0.741361 | −0.696467 |
| C | 1.759211 | −1.564322 | −0.696467 |
| C | −1.497741 | 0.006808 | −1.824018 |
| H | −3.306177 | −0.536316 | −0.724375 |
| H | −2.098740 | −1.823680 | −0.824139 |
| H | −1.852678 | 1.043890 | −1.835880 |
| H | −1.749891 | −0.435376 | −2.794653 |
| C | 0.742975 | −1.300486 | −1.824018 |
| C | 0.754767 | 1.293678 | −1.824018 |
| H | 2.628724 | −0.905722 | −0.824139 |
| H | 2.117552 | −2.595076 | −0.724375 |
| H | 1.251992 | −1.297762 | −2.794653 |
| H | 0.022304 | −2.126411 | −1.835880 |
| H | 1.188625 | 3.131392 | −0.724375 |
| H | −0.529983 | 2.729403 | −0.824139 |
| H | 0.497898 | 1.733138 | −2.794653 |
| H | 1.830374 | 1.082521 | −1.835880 |
| N | 0.000000 | 0.000000 | 2.632344 |

| | | | |
|---|---|---|---|
| C | 0.000000 | 0.000000 | 3.772148 |
| H | 0.000000 | 0.000000 | 4.840009 |
| O | −1.740243 | −0.353226 | 0.581263 |
| O | 0.564219 | 1.683708 | 0.581263 |
| O | 1.176024 | −1.330482 | 0.581263 |

$\mathbf{2}_{Ge3}$ ··· NCH :

| | | | |
|---|---|---|---|
| Ge | 0.000000 | 0.000000 | 0.366261 |
| C | 0.000000 | 0.000000 | −1.577266 |
| C | 2.670599 | 0.160338 | 0.108966 |
| C | −1.474156 | 2.232637 | 0.108966 |
| C | −1.196443 | −2.392975 | 0.108966 |
| C | −1.055343 | 0.997900 | −2.078303 |
| H | −1.633802 | 3.226916 | 0.531447 |
| H | −2.425342 | 1.685707 | 0.187599 |
| C | −0.336535 | −1.412903 | −2.078303 |
| C | 1.391878 | 0.415004 | −2.078303 |
| H | −0.247194 | −2.943262 | 0.187599 |
| H | −1.977690 | −3.028372 | 0.531447 |
| H | 3.611492 | −0.198544 | 0.531447 |
| H | 2.672536 | 1.257554 | 0.187599 |
| C | 2.569143 | −0.241720 | −1.356841 |
| C | −1.493907 | −2.104083 | −1.356841 |
| C | −1.075236 | 2.345803 | −1.356841 |
| H | −2.405734 | −1.500170 | −1.408663 |
| H | −1.713932 | −3.051247 | −1.857447 |
| H | 2.502052 | −1.333342 | −1.408663 |
| H | 3.499423 | 0.041315 | −1.857447 |
| H | −1.785492 | 3.009932 | −1.857447 |
| H | −0.096318 | 2.833511 | −1.408663 |
| H | 0.548152 | −2.053036 | −1.980078 |
| H | −0.547652 | −1.342914 | −3.155305 |
| H | 1.436824 | 0.197177 | −3.155305 |
| H | 1.503906 | 1.501231 | −1.980078 |
| H | −2.052057 | 0.551805 | −1.980078 |
| H | −0.889172 | 1.145737 | −3.155305 |
| N | 0.000000 | 0.000000 | 3.564573 |
| C | 0.000000 | 0.000000 | 4.711109 |
| H | 0.000000 | 0.000000 | 5.777885 |
| O | −1.147113 | −1.218437 | 0.902718 |
| O | 1.628754 | −0.384211 | 0.902718 |
| O | −0.481640 | 1.602648 | 0.902718 |

$\mathbf{2}_{Sn2}$ :

| | | | |
|---|---|---|---|
| Sn | 0.000000 | 0.000000 | 0.771849 |
| C | 0.000000 | 0.000000 | −1.304658 |
| C | 0.000000 | 2.398235 | −0.441957 |
| C | −2.076933 | −1.199118 | −0.441957 |
| C | 2.076933 | −1.199118 | −0.441957 |
| C | −1.475268 | −0.320110 | −1.567437 |
| H | −3.158382 | −1.277894 | −0.567570 |
| H | −1.656206 | −2.211378 | −0.512897 |
| H | −2.038486 | 0.619859 | −1.595392 |
| H | −1.639937 | −0.823792 | −2.527337 |
| C | 1.014857 | −1.117565 | −1.567437 |
| C | 0.460411 | 1.437675 | −1.567437 |
| H | 2.743212 | −0.328628 | −0.512897 |
| H | 2.685879 | −2.096292 | −0.567570 |
| H | 1.533393 | −1.008331 | −2.527337 |
| H | 0.482429 | −2.075311 | −1.595392 |
| H | 0.472503 | 3.374186 | −0.567570 |
| H | −1.087006 | 2.540005 | −0.512897 |
| H | 0.106543 | 1.832123 | −2.527337 |
| H | 1.556057 | 1.455451 | −1.595392 |
| O | 0.334309 | 1.940524 | 0.875592 |
| O | −1.847698 | −0.680742 | 0.875592 |
| O | 1.513388 | −1.259782 | 0.875592 |

$\mathbf{2}_{Sn3}$ :

| | | | |
|---|---|---|---|
| Sn | 0.000000 | 0.000000 | 0.762114 |
| C | 0.000000 | 0.000000 | −1.355621 |
| C | 2.596330 | 1.095304 | 0.366899 |
| C | −2.246726 | 1.700835 | 0.366899 |
| C | −0.349604 | −2.796140 | 0.366899 |
| C | −1.266639 | 0.731294 | −1.818385 |
| H | −2.731850 | 2.610371 | 0.730805 |
| H | −3.032263 | 0.937658 | 0.257859 |
| C | 0.000000 | −1.462589 | −1.818385 |
| C | 1.266639 | 0.731294 | −1.818385 |
| H | 0.704096 | −3.094846 | 0.257859 |
| H | −0.894722 | −3.671037 | 0.730805 |
| H | 3.626572 | 1.060666 | 0.730805 |
| H | 2.328167 | 2.157188 | 0.257859 |
| C | 2.533518 | 0.427530 | −1.009895 |
| C | −0.896507 | −2.407856 | −1.009895 |
| C | −1.637011 | 1.980325 | −1.009895 |
| H | −1.893454 | −1.969996 | −0.871558 |
| H | −1.041409 | −3.330101 | −1.578964 |
| H | 2.652794 | −0.654781 | −0.871558 |
| H | 3.404657 | 0.763164 | −1.578964 |
| H | −2.363248 | 2.566937 | −1.578964 |
| H | −0.759340 | 2.624777 | −0.871558 |
| H | 1.023595 | −1.854958 | −1.786766 |
| H | −0.297283 | −1.473708 | −2.877155 |
| H | 1.424910 | 0.479399 | −2.877155 |
| H | 1.094643 | 1.813938 | −1.786766 |
| H | −2.118238 | 0.041020 | −1.786766 |
| H | −1.127627 | 0.994309 | −2.877155 |
| O | −1.316466 | 1.309550 | 1.362925 |
| O | −0.475871 | −1.794868 | 1.362925 |
| O | 1.792336 | 0.485318 | 1.362925 |

$\mathbf{2}_{Sn2}$ ··· NCH :

| | | | |
|---|---|---|---|
| Sn | 0.000000 | 0.000000 | 0.437276 |
| C | 0.000000 | 0.000000 | −1.666336 |
| C | 0.493288 | 2.363951 | −0.850721 |
| C | −2.293885 | −0.754776 | −0.850721 |
| C | 1.800597 | −1.609175 | −0.850721 |
| C | −1.501797 | 0.005970 | −1.944738 |
| H | −3.363415 | −0.579547 | −0.991131 |
| H | −2.118379 | −1.833559 | −0.972966 |
| H | −1.852032 | 1.045185 | −1.949241 |
| H | −1.756200 | −0.424313 | −2.921809 |
| C | 0.745728 | −1.303579 | −1.944738 |
| C | 0.756068 | 1.297609 | −1.944738 |
| H | 2.647098 | −0.917790 | −0.972966 |
| H | 2.183610 | −2.623030 | −0.991131 |
| H | 1.245566 | −1.308757 | −2.921809 |
| H | 0.020859 | −2.126500 | −1.949241 |
| H | 1.179806 | 3.202577 | −0.991131 |
| H | −0.528719 | 2.751349 | −0.972966 |
| H | 0.510634 | 1.733070 | −2.921809 |
| H | 1.831173 | 1.081314 | −1.949241 |
| N | 0.000000 | 0.000000 | 2.755279 |
| C | 0.000000 | 0.000000 | 3.894930 |
| H | 0.000000 | 0.000000 | 4.963941 |
| O | −1.953351 | −0.363762 | 0.472394 |
| O | 0.661648 | 1.873532 | 0.472394 |
| O | 1.291702 | −1.509770 | 0.472394 |

$\mathbf{2}_{Sn3}$ ··· NCH :

| | | | |
|---|---|---|---|
| Sn | 0.000000 | 0.000000 | 0.494414 |
| C | 0.000000 | 0.000000 | −1.652695 |
| C | 2.867790 | 0.361658 | −0.037083 |
| C | −1.747100 | 2.302750 | −0.037083 |
| C | −1.120690 | −2.664408 | −0.037083 |
| C | −1.002016 | 1.050476 | −2.141822 |
| H | −1.865458 | 3.320294 | 0.350742 |
| H | −2.758448 | 1.915919 | −0.235399 |
| C | −0.408731 | −1.393009 | −2.141822 |
| C | 1.410747 | 0.342533 | −2.141822 |
| H | −0.280010 | −3.346845 | −0.235399 |
| H | −1.942730 | −3.275681 | 0.350742 |
| H | 3.808188 | −0.044613 | 0.350742 |
| H | 3.038458 | 1.430927 | −0.235399 |
| C | 2.544719 | −0.324788 | −1.361685 |
| C | −1.553634 | −2.041397 | −1.361685 |
| C | −0.991085 | 2.366185 | −1.361685 |
| H | −2.345844 | −1.307707 | −1.161795 |
| H | −2.007806 | −2.831364 | −1.966424 |
| H | 2.305430 | −1.377707 | −1.161795 |
| H | 3.455936 | −0.323129 | −1.966424 |
| H | −1.448130 | 3.154493 | −1.966424 |
| H | 0.040414 | 2.685414 | −1.161795 |
| H | 0.455136 | −2.067871 | −2.102547 |
| H | −0.672599 | −1.300339 | −3.206422 |
| H | 1.462426 | 0.067682 | −3.206422 |

| | | | |
|---|---|---|---|
| H | 1.563260 | 1.428095 | −2.102547 |
| H | −2.018396 | 0.639776 | −2.102547 |
| H | −0.789827 | 1.232657 | −3.206422 |
| N | 0.000000 | 0.000000 | 2.964748 |
| C | 0.000000 | 0.000000 | 4.105992 |
| H | 0.000000 | 0.000000 | 5.173103 |
| O | −0.766723 | −1.747247 | 0.972056 |
| O | 1.896522 | 0.209622 | 0.972056 |
| O | −1.129799 | 1.537625 | 0.972056 |
